# Impact on Human Health of Microorganisms Present in Fermented Dairy Products: An Overview

**DOI:** 10.1155/2015/412714

**Published:** 2015-03-09

**Authors:** María Fernández, John Andrew Hudson, Riitta Korpela, Clara G. de los Reyes-Gavilán

**Affiliations:** ^1^Instituto de Productos Lácteos de Asturias, Consejo Superior de Investigaciones Científicas (IPLA-CSIC), Paseo Río Linares s/n, Villaviciosa, 33300 Asturias, Spain; ^2^Food Safety Programme, ESR-Christchurch Science Centre, Christchurch 8540, New Zealand; ^3^Food and Environment Safety Programme, The Food and Environment Research Agency, Sand Hutton, York YO41 1LZ, UK; ^4^Medical Nutrition Physiology Group, Pharmacology, Institute of Biomedicine, University of Helsinki, 00014 Helsinki, Finland

## Abstract

Fermented dairy products provide nutrients in our diet, some of which are produced by the action of microorganisms during fermentation. These products can be populated by a diverse microbiota that impacts the organoleptic and physicochemical characteristics foods as well as human health. Acidification is carried out by starter lactic acid bacteria (LAB) whereas other LAB, moulds, and yeasts become dominant during ripening and contribute to the development of aroma and texture in dairy products. Probiotics are generally part of the nonstarter microbiota, and their use has been extended in recent years. Fermented dairy products can contain beneficial compounds, which are produced by the metabolic activity of their microbiota (vitamins, conjugated linoleic acid, bioactive peptides, and gamma-aminobutyric acid, among others). Some microorganisms can also release toxic compounds, the most notorious being biogenic amines and aflatoxins. Though generally considered safe, fermented dairy products can be contaminated by pathogens. If proliferation occurs during manufacture or storage, they can cause sporadic cases or outbreaks of disease. This paper provides an overview on the current state of different aspects of the research on microorganisms present in dairy products in the light of their positive or negative impact on human health.

## 1. The Microbial World Diversity in Fermented Dairy Products

Fermented dairy products are an important part of our diet and can contain a diverse microbiota. Lactic acid bacteria (LAB) are the main players during milk fermentation, converting lactose to lactic acid, which results in an increased acidity that makes growth conditions of microorganisms other than LAB increasingly unfavourable. The LAB involved in fermented dairy processing belong to diverse microbial groups that are characterized by different nutritional, metabolic, and culture requirements as well as different technological properties. The most common LAB present in milk includes species belonging to the genera* Lactobacillus*,* Streptococcus*,* Leuconostoc*,* Enterococcus,* and* Lactococcus* [1].

*Lactococcus lactis* ssp.* lactis* and* Lactococcus lactis* ssp.* cremoris*, in particular, are primarily known because of their role as starter cultures for the cheese industry. The genus* Lactobacillus* currently consists of 174 different species. Lactobacilli play two main roles in fermented dairy products, as starters or as secondary microbiota.* Lactobacillus delbrueckii* ssp.* bulgaricus* and* Lactobacillus delbrueckii* ssp.* lactis* are used worldwide as starters in yoghurt production. In contrast, other lactobacilli initially present in raw milk increase in number during the manufacture of dairy products and can become particularly dominant during cheese ripening [2]. These populations, which are often referred to as nonstarter LAB (or NSLAB), are able to carry out proteolysis and lipolysis, subsequently producing many end products that contribute to the development of flavour and texture of cheeses [3]. The species more frequently involved include* Lactobacillus helveticus*,* Lactobacillus casei*,* Lactobacillus paracasei, Lactobacillus plantarum/paraplantarum, Lactobacillus rhamnosus, Lactobacillus curvatus, Lactobacillus brevis, Lactobacillus sake, Lactobacillus pentosus, Lactobacillus acidophilus, Lactobacillus reuteri, Lactobacillus johnsonii, Lactobacillus crispatus, Lactobacillus fermentum, Lactobacillus buchneri,* and* Lactobacillus gasseri*. While the analysis of the presence and levels of these species in food products can be underestimated with the use of culture-dependent methods, the development of culture-independent techniques for the study of microbial communities, such as PCR-DGGE, PCR-TTGE, qPCR, 16S rRNA gene sequencing, and metagenomic approaches, is contributing to a deeper knowledge of the fermented dairy products microbiota. Although many streptococcal genera are pathogenic,* Streptococcus thermophilus* carries a “GRAS” status [4].* S. thermophilus* is a thermophilic LAB widely used as starter culture in the manufacture of dairy products, notably in the yoghurt production, and is considered as the second most important industrial dairy starter after* La. lactis*. Enterococci are the most controversial group of food-associated LAB and they could act either as starter cultures, probiotics, spoilage, or pathogenic organisms depending on the strain considered [5].* Leuconostoc*, in particular the species* Leuconostoc mesenteroides* and* Leuconostoc pseudomesenteroides*, have the ability to produce CO_2_ which is responsible for the eye formation in some types of cheeses [6]. Other microbial groups comprising Gram-positive and Gram-negative bacteria, as well as yeasts and moulds, also contribute to the organoleptic and physicochemical properties of dairy products. In this regard, Gram-positive bacteria like* Corynebacterium* spp.,* Arthrobacter* spp., and* Brevibacterium* are essential in smear-ripened cheeses.* Propionibacterium freudenreichii* subsp.* shermanii* carries out the propionic fermentation through the conversion of lactic acid formed by acidifying bacteria to acetate, propionate, and CO_2_, the latter being responsible of the eye formation in Swiss-type and other cheeses.

Bifidobacteria represent an important group of nonstarter microorganisms that are included in some dairy products, mainly fermented milks, because of the health-promoting properties attributed to some of them. Although they usually have a considerably slower growth-rate than starter cultures, their proliferation will contribute to increase levels of lactate and acetate in final products.

Regarding undesirable microorganisms in dairy products, special attention should be focused on the spore-former bacteria which are important contaminants in the dairy industry. Thus, microorganisms belonging to the genus* Clostridium*, such as* Clostridium tyrobutyricum* or* Clostridium butyricum*, are considered the main organisms responsible for the late-blowing of cheese [7]. Pathogenic clostridia will be commented on below. The presence of contaminating Gram-negative bacteria, mainly enterobacteria, is rather common in dairy foods, sometimes reaching levels up to 10^6^-10^7^ CFU g^−1^ in cheeses and they can contribute to a worsening of sensory quality of dairy products [8].

Yeast and moulds are important microbial populations in dairy products, especially in some types of cheeses. As with bacteria, the development of culture independent DNA-based analytical methods has allowed detection of genera and species not previously found in dairy environments, such as* Torrubiella* and* Malassezia* [9]. In cheese, yeasts and moulds play a key role in the development and enhancement of texture and flavour through the activity of some microbial extracellular enzymes in the food matrix. The yeast species most frequently found in dairy products include* Kluyveromyces lactis*,* Debaryomyces hansenii, Candida* spp.,* Geotrichum candidum,* and* Yarrowia lipolytica*. Among moulds* Penicillium*,* Geotrichum*,* Aspergillus*,* Mucor,* and* Fusarium* are the most common genera [10].

## 2. Beneficial and Toxic Compounds Released by LAB, Yeasts, and Moulds during Fermentation

Some health-promoting properties of fermented dairy products are due to the synthesis or to the release from the food matrix of bioactive compounds as a result of the metabolic activity of LAB, propionibacteria, yeast, and moulds. Worth mentioning are among others, conjugated linoleic acid (CLA), exopolysaccharides (EPS), bioactive peptides, vitamins, gamma-aminobutyric acid (GABA), and oligosaccharides [11].

Although milk contains vitamins, fermentation by LAB often leads to the enrichment of some of them, as it is the case for vitamin B_12_, folic acid, and biotin produced by propionibacteria [12] or the higher synthesis of folate in milk fermented with some LAB with respect to nonmilk complex culture media [13]. CLA is a native component of milk fat. Its content can be increased in fermented milk through bioconversion of unsaturated fatty acids such as linoleic and linolenic acids by different LAB [14, 15]. The functionality of CLA has been well documented with respect to its anti-inflammatory [16], antiatherogenic, and antioxidant properties [17].

Bioactive peptides are specific fragments of milk proteins that are released by proteolytic activity from caseins predominantly and also from whey proteins. Antihypertensive, antimicrobial, antioxidative, and immune-modulatory activities have been described for peptides released as a result of the activity of LAB in fermented milk products [18]. In general, their bioactive characteristics are based on the specific amino acid sequence and chain length (generally from two to twenty residues) as well as on their resistance to hydrolysis. The most studied mechanism of bioactive peptides is the antihypertensive action displayed by the inhibition of the angiotensin-I-converting enzyme (ACE; peptidyldipeptide hydrolase, EC 3.4.15.1) which regulates blood pressure [19]. Some* Lactobacillus*-fermented milks and cheeses with added probiotic lactobacilli revealed ACE-inhibitory activity [20, 21]. GABA is another compound with blood pressure repressing properties; it has been demonstrated to be produced in fermented milk by* Lb. casei* Shirota and* La. lactis* through transformation of glutamic acid derived from milk proteins [22]. Bacteriocins are also among the beneficial peptides intrinsically synthesized by some LAB during milk fermentation and their usefulness in preventing growth of undesirable and pathogenic microorganisms during milk fermentation has been commented on above.

Galactooligosaccharide (GOS) synthesis by LAB is due to a transgalactosylation side-line activity by *β*-galactosidase on lactose, the main sugar of milk. GOS have recognized prebiotic effect on intestinal microbiota, promoting selective growth of bifidobacteria [23, 24]. EPS are complex extracellular carbohydrate polymers produced by some microorganisms. They can protect the producer strains against environmental adverse factors and some of them positively interact with the colonic microbiota and with the immune system of the host [25, 26].

Special mention is deserved of bioactive peptide components of proteins secreted by LAB and probiotic bacteria. This is the case of the enriched serine/threonine peptide derived from one of the main extracellular proteins produced by* Lb. plantarum*, which displayed immunomodulatory properties after being released during digestion [27].

Although the metabolic activity of microorganisms during dairy fermentation yields mostly beneficial compounds, in some cases metabolic activities result in the release of toxic substances for the consumer. Two types of toxic compounds have been identified in dairy products, mycotoxins produced by some fungi, and biogenic amines (BA) mainly due to the metabolic activity of some LAB.

Mycotoxins are chemical hazards synthesized primarily by three genera of filamentous fungi:* Aspergillus*,* Fusarium,* and* Penicillium* [28]. They are termed secondary metabolites, because they are not essential for normal growth and development. Although fungi can collectively produce hundreds of mycotoxins, only trichothecenes, fumonisins, and zearalenone (produced by* Fusarium* species) and aflatoxins, ochratoxins, and patulin (produced by* Aspergillus* and* Penicillium* species) are of note from a health point of view [28]. These secondary metabolites are products of multistep biochemical pathways. The genes encoding the synthase, the modifying enzymes, the transporters, and the transcriptional regulators are typically located next to one another in a gene cluster [29]. In milk and dairy products mycotoxins mainly come from feed contaminated either in the field or during drying and storage. One of the most economically important mycotoxins worldwide is aflatoxin. This polyketide produced mainly by* Aspergillus flavus* and* Aspergillus parasiticus* is a potent carcinogen [28]. Aflatoxin M1, that results from the metabolic conversion of aflatoxin B1, can occur in milk and milk products from animals consuming feedstuffs contaminated with B1 aflatoxins. Aflatoxin-producing* Aspergillus* can contaminate grain before harvest or during storage; favourable conditions of temperature, humidity, and mechanical kernel damage during harvesting, among other factors, may favour the active production of aflatoxin B1 in contaminated grains. Aflatoxin B1 is transformed to aflatoxin M1 in the liver of lactating animals and is excreted by the mammary gland. The potential occurrence of mycotoxins in dairy products and mainly in milk makes them a particular risk for humans because of their negative effects for adults and, especially, children [30].

The other toxic compounds mainly associated to the metabolism of some bacteria are BA. These are low-molecular weight nitrogenous organic bases with biological activity, mainly synthesized by decarboxylation of the corresponding amino acids. The most important and frequent BA found in dairy products are histamine, tyramine, and putrescine, which are produced by decarboxylation of histidine, tyrosine, and ornithine, respectively. Putrescine can also be synthesized by deimination of agmatine. Cadaverine (originating from lysine decarboxylation) is found less frequently in dairy products [31]. BA are naturally present in vegetables, animals, and humans, being involved in important biological processes. Many bacteria of different genera and species have the capacity to produce BA. Gram-negative bacteria (mainly Enterobacteriaceae) that can be present in milk are able to produce histamine, putrescine, and cadaverine [32–34]. However, the main BA producers in dairy products are mostly LAB of the genera* Enterococcus*,* Lactobacillus*,* Leuconostoc*,* Lactococcus,* and* Streptococcus* [35–39]. These bacteria can be (i) present in milk, (ii) introduced by contamination throughout the entire process of cheese production, (iii) and even part of starter or adjunct cultures [40]. Among the fermented dairy products, cheeses can have the highest BA concentrations, because of their complex microbiota and the availability of precursor amino acids from casein proteolysis. In fact, BA concentrations up to 2,000 mg per kg of cheese have been reported [41, 42]. The intake of such contaminated foods could cause serious health problems. For this reason, during the recent past the metabolic pathways involved in the synthesis of these compounds and the environmental conditions favouring their accumulation in foods have been studied in depth [40], in parallel with the development of reliable detection methods either for BA or for the microbial BA producers [43, 44].

A general picture of beneficial and detrimental compounds produced by microorganisms present in dairy products is indicated in Table 2.

## 3. Probiotics and Mechanisms of Beneficial Action

Probiotics are live microorganisms which confer a health benefit on the host when administered in adequate amounts [45]. The most commonly investigated and commercially available probiotics are mainly microorganisms from species of the genera* Lactobacillus* and* Bifidobacterium*. In addition, several others such as* Propionibacterium*,* Streptococcus*,* Bacillus*,* Enterococcus*,* Escherichia coli*, and yeasts are also used [46, 47]. Probiotics must be able to survive in the gastrointestinal tract and be resistant to gastric juices and bile. They should exert benefits to the host through their activity in the human body. In order to confer health benefits, they should be nonpathogenic and nontoxic and provide protection against pathogenic microorganisms by means of multiple mechanisms [45]. In addition, probiotics should be lacking transferable antibiotic resistance genes. Different bacterial strains of the same genus and species may exert different effects on the host. The most promising health effects of probiotics in human intervention studies include amelioration of acute diarrhoea in children, reduction of the risk of respiratory tract infections, relief of children's milk allergy/atopic dermatitis, and alleviation of irritable bowel syndrome. Probiotics may exert their beneficial health effects by normalization of the host's microbiota, by inhibition of pathogens, by interaction with the immune system of the host, and through their own metabolic activity. Probiotics may also enhance the resilience of microbiota against detrimental outside factors. However, the molecular mechanisms behind the effects are largely unknown.

### 3.1. Inhibition of Pathogens

Clinical and animal studies have demonstrated that specific probiotics are effective in alleviating infections, but the mechanisms of action are not completely understood. Additionally, beneficial properties and efficacy can vary considerably among different strains from the same species. Possible mechanisms of probiotic action include (1) hindering the adsorption, (2) cell internalization of the pathogen, (3) production of metabolites and substances with a direct effect on the pathogen, and (4) crosstalk (immunomodulation) with the cells in establishing the protection [47, 48]. The possible mechanisms by which probiotics may act against infections are presented in Table 3.

The gastrointestinal and respiratory tracts are covered by mucosal epithelial surfaces which are constantly exposed to numerous microorganisms and serve as primary ports of entry for most infectious viruses. Pathogen attachment to a host cell is the first step in the disease process, and, therefore, interruption of this attachment could be beneficial to the host. Probiotic bacteria may bind directly to the pathogen and inhibit pathogen attachment to the host cell receptor. For instance, there is evidence that,* in vitro*, specific strains of lactobacilli and bifidobacteria are able to bind and inactivate rotavirus [49] and vesicular stomatitis virus [50]. In addition, adhesion of probiotics on the epithelial surface [51–53] may block pathogen attachment by steric hindrance, cover receptor sites in a nonspecific manner, or inhibit binding of pathogens to specific carbohydrate receptors. Luminal secretions (mucus, glycolipids, and protective peptides) and antimicrobial peptides (defensins) may also protect epithelial cells from infections. Intestinal mucins may bind to pathogens through specific mucin-bacterial/viral interaction and inhibit their adherence to the epithelial cells [54]. Probiotics may induce mucosal regeneration by increasing mitosis rate in the small intestine and increasing the numbers of cells in the villi [55, 56]. They can also promote intestinal epithelial homeostasis via soluble proteins [57]. Probiotics also show direct activity against pathogens by producing antimicrobial substances such as organic acids, hydrogen peroxide, diacetyl, short chain fatty acids, biosurfactants, and bacteriocins. It is widely known that intestinal permeability increases in gastrointestinal infections, as pathogens attach to cell receptors below the tight junctions on the basolateral membrane, thus modifying tight junctions and disturbing the barrier. A possible mechanism of probiotics beneficial action is the reinforcement of gut defence barrier by normalizing permeability and disturbed gut microbial ecology [47, 58, 59].

### 3.2. Interaction with the Immune System

An optimally functioning immune system is important for the maintenance of physiological integrity and health. The immune system provides defence against infections caused by pathogenic microorganisms. It also modulates our health and well-being in many ways sometimes by up- or downregulating the defence system. An effectively functioning immune system is fundamental for protection against infectious diseases. One possible probiotic mechanism against infections could be the stimulation of the gut immune system. In the gut epithelial cells, probiotics can be recognized by toll-like receptors [60–63]. Probiotics may, therefore, modulate cytokine expression patterns through epithelial cells [64] and/or through macrophages and dendritic cells [65–70]. Many experimental studies* in vitro* show that certain strains of probiotics are capable of providing protection against infections by stimulating antiviral, cytokine, and chemokine responses in gastrointestinal and respiratory epithelial cells or immune cells. Administration of lactobacilli to mice may affect respiratory infections by reducing virus titre in the lungs and increasing survival rate of the animals via stimulating innate immune responses [47, 71].

## 4. Strategies to Improve Viability and Functionality of Probiotics in Fermented Dairy Products and the Gastrointestinal Tract

Probiotics are generally added as adjunct cultures in fermented dairy products. Their viability in foods should ensure the minimum daily dose able to provide the health benefits attributed to the specific functional food product in which they are included. However, probiotics often show poor survival in the food matrix, due to factors such as low pH, oxygen content, temperature, and the presence of other microorganisms. In addition, probiotics should remain viable at sufficient levels through the gastrointestinal transit in order to arrive alive to the site of action, the intestine. During digestion, they have to face different harsh physiological barriers, including digestive enzymes, the acidic pH of the stomach, and bile salts in the intestine and then compete with members of the resident intestinal microbiota for scarce fermentable substrates. In addition, not only the viability but also the maintenance of the metabolic activity and the beneficial properties of strains are important [46].

Some strategies targeting the food product and/or the composition of starter cultures have been used to improve viability of microorganisms in fermented dairy products. The selection and combination of appropriate LAB strains [72, 73], the control of the final pH and postacidification phenomenon by different approaches [74, 75], or the addition of protectors and oxygen scavengers [6, 76] are some examples.

Other strategies affecting the microorganism itself are useful to increase survival in the food matrix and during the gastrointestinal transit. For example, the selection of EPS-producing probiotics could be an appropriate way to obtain strains with adequate viability, since these polymers can act as protectors of the producing bacteria, contributing to their viability [77, 78]. Resistant derivatives to technological or physiological conditions are easy to obtain by exposing the probiotic to sublethal stressing factors (freezing, heat, drying, oxygen, acid, bile, NaCl, etc.). Usually, the resistant microorganisms present a stable phenotype with higher viability, but they often develop cross-resistances to other stresses [79]. Adaptation to stress may also influence physiological characteristics of microorganisms that could hence impact technological and sensory aspects as well as probiotic-related properties [80–82]. Gene modification is another way to increase stress tolerance. However, the use of such genetically modified microorganisms is limited by current regulation in several countries [83, 84].

Addition of some food ingredients to food could enhance survival of probiotics, as is the case of prebiotics. These can be defined as “a selectively fermented ingredient that results in specific changes in the composition and/or activity of the gastrointestinal microbiota, thus conferring benefits upon host health” [85]. Most prebiotics are complex carbohydrates from plant origin. Probiotics have been employed in combination with prebiotics (synbiotics) to improve their viability; prebiotics often act as entrapping matrices during the gastrointestinal transit, further releasing the microorganism in the intestine and then serving as fermentable substrates [86]. Microencapsulation of probiotics on different materials has been also used to enhance the viability [87].

## 5. Opportunistic and Pathogenic Microorganisms and Mechanisms of Detrimental Action in the Host

Gram-positive bacteria associated with food poisoning comprise mainly nonsporulating microorganisms from the genera* Staphylococcus* and* Listeria,* as well as sporulating* Clostridium tertium*,* Clostridium perfringens, Clostridium botulinum,* and members of the* Bacillus cereus* group [88]. Some Gram-negative bacteria contaminating dairy foods are considered as indicators of poor hygiene and may constitute a health risk if pathogenic species are present. These include the species* E. coli*,* Pseudomonas aeruginosa*,* Klebsiella pneumoniae*, and* Citrobacter freundii* and the genera* Enterobacter*,* Proteus*,* Psychrobacter*,* Halomonas,* and* Serratia *[89]. Specific pathogens are mainly particular enterotoxigenic* E. coli* pathotypes and* Salmonella.*

The proportion of foodborne disease outbreaks and sporadic cases that can be attributed to the consumption of dairy products was approximately 4–7% of outbreaks in the USA from 1998 to 2008 [90] and in 2009-2010 this figure was 13% [91]. Only a proportion of these would be attributable to fermented dairy products. In the EU, cheese was identified as the vehicle of transmission in 41 of 763 (5.4%) outbreaks and other dairy products (excluding milk) and in only 4 (0.5%) during 2012 [92]. Inspection of the data shows that many of these outbreaks are, in fact, associated with coagulated dairy products that have not been fermented but produced by direct acidification. The behaviour of pathogens is different in cheeses produced with or without a starter culture [93]. Of six dairy-associated outbreaks of listeriosis recorded in the USA from 1998 to 2008, four were caused by Mexican-style queso fresco/queso blanco which are soft cheeses produced without starter cultures [94].

Raw (unpasteurized) milk can contain a variety of bacterial pathogens which may cause disease if not eliminated during production [95]. Disease can be caused by two major mechanisms: infection by the organism or ingestion of preformed toxin.* Listeria monocytogenes *[96],* E. coli* O157:H7, and other shiga toxigenic* E. coli* (STEC) [97] can cause significant clinical outcomes. Around 20–30% of listeriosis cases are fatal while STEC infections can cause kidney failure and, more rarely, death, especially in young people. Salmonellosis is usually a diarrhoeal disease, while brucellosis is a systemic infection causing symptoms such as fever, fatigue, and myalgia. In contrast, both* Staphylococcus aureus* and* C. botulinum* can grow in foods to form toxins [98, 99]. Staphylococcal enterotoxin can result in emesis when ingested, while botulinum toxin can result in paralysis and death in an estimated 17.3% of domestically acquired foodborne cases of botulism in United States [100].

In cheesemaking using raw milk, initial production steps can involve periods where the milk is held at >30°C, temperatures which may allow contaminating bacteria to proliferate. However, in general, subsequent steps result in inactivation of bacterial pathogens. The use of a starter culture is critical because of the resulting low pH concomitant with the production of lactic acid [101]. During fermentation, milk and curd may rapidly reach a pH at which pathogens will not grow and subsequently their levels will decline as long as the pH remains low. The potential for pathogens to survive manufacture and ripening to contaminate the retail product made from raw milk depends mainly on (1) the initial levels of the pathogen, (2) growth and entrapment in the curd during manufacture, (3) the rate of microbial population decrease during ripening, (4) antagonistic activity of LAB present in milk or added as starters, (5) physicochemical parameters, such as pH, salt content, and water activity, and (6) the length of ripening.

In cheeses which are mould-ripened or bacteria smear-ripened (e.g., smear cheeses), the fungi or bacteria used to achieve the particular characteristics of the product cause a rise in the pH during ripening and so potentially allowing surviving pathogens to grow. The fate of various pathogens in cheese production has been reviewed [102].

Pasteurisation is the common method to eliminate pathogens from milk prior to the manufacture of dairy products, and so when contamination occurs it is a result of poor hygiene practices postpasteurisation or pasteurisation failure. While there has been much public debate about the relative merits of consuming dairy products made with raw milk versus pasteurised milk, when consumption volumes are considered, raw milk products cause a disproportionately large proportion of cases of foodborne disease compared to those made with pasteurised milk [91, 103, 104].

As a whole, despite the overall excellent safety record of fermented dairy products, outbreaks and incidents of disease still can result from their consumption [105–112].  Table 1 gives some examples of outbreaks, the pathogens that caused them, and the reasons why they occurred.

## 6. Strategies for Counteracting Pathogens and Harmful Microorganisms in Fermented Dairy Products

The most common approach to guarantee the safety of fermented dairy products is to ensure that the milk used in their manufacture is pathogen-free (or contains an acceptably low level of some pathogens like* S. aureus*) followed by the prevention of recontamination during production, distribution, and retail sale. With current technology, pathogen-free raw milk is difficult to produce, but using food quality milk from a source that submits animals to a strict pathogen testing regime and has good hygiene practices in place may help to meet this goal.

Pasteurisation (usually, the exposure to 72°C for 15 seconds, or 63°C for 30 min) is considered to be sufficient to remove bacterial pathogens from milk intended for the use in fermented dairy products. An alternative to pasteurisation of milk, which is implemented in several countries, is to age cheeses made from raw milk for 60 days as a minimum. However, this has been shown to be ineffective under some circumstances such as when pathogenic strains are resistant to low pH or in postprocessing contamination of surface-mold-ripened cheeses in which a rise of pH occurs during maturation [113, 114].

Alternatives to pasteurisation have been sought in order to produce safe milk for processing yet not producing the perceived organoleptic changes resulting from pasteurisation. Some examples of this are ultrahigh pressure treatment [115], pulsed electric field (PEF) technology, and ultrasonication [116]. High hydrostatic pressure has been applied to both milk used to make cheese [117] and cheese itself [118] where significant reductions in* S. aureus* were recorded. PEF is not particularly effective with bacterial spores but kills vegetative cells, typically by 4-5 log_10_ CFU/mL, through the production of pores in bacterial membranes. There may also be an improved curd quality in cheese made using PEF milk. Ultrasonication works primarily by cavitation which causes shear stress and physical damage to cells, but the effects are only significant at temperatures above 50°C. It can be used in combination with other physicochemical treatments [115].

There are also a number of nonphysicochemical measures which could broadly be termed biocontrol, including the use of bacteriophages, bacteriocins/protective cultures, and naturally-occurring chemicals, such as essential oils. Bacteriophages (phages) are bacterial viruses. They have been shown to control* Salmonella* in cheddar cheese production [119],* S. aureus* in fresh and hard cheese production [120], and* E. coli* O157 in fermented milk production [121]. After 90 days of storage levels of* Salmonella* were consistently 2-3 log_10_ CFU g^−1^ higher in untreated cheeses compared to those in phage-treated cheeses. Control of* L. monocytogenes* by phages has been similarly reported for smear-ripened soft cheeses [122]. The cheese was ripened at 14°C for 16 days, packaged, and then stored for five more days at 6°C. The levels of* L. monocytogenes* reached 10^5^ CFU cm^−2^ in untreated cheeses at 16 days and >10^7^ CFU cm^−2^ by day 21. Application of the phage preparation eliminated* L. monocytogenes* and no further growth occurred during storage. Similar results have been reported elsewhere [123]. Starter and nonstarter LAB can act as a protective culture [124], inhibiting the growth of pathogens through competition (pH reduction, production of hydrogen peroxide, etc.) and/or by the production of bacteriocins [101]. Bacteriocins are a heterogeneous group of antimicrobial peptides that inhibit the growth of other bacteria. These compounds generally display action on a narrow range of organisms. Whereas some of them only act against other LAB, others are also able to inhibit the growth of some foodborne pathogenic bacteria [125], serving as natural biopreserving agents in fermented dairy products. Nisin, a commercially available bacteriocin, has found use in the prevention of the outgrowth of spores, particularly those of* Clostridium *species [101, 125], allowing flexibility in the formulation of dairy products such as processed cheese. NSLAB producing bacteriocins can be used singly and in combination with high pressure to kill pathogens in cheese [126].

A novel idea is to use plant-derived essential oils to control pathogens. For example, oregano and thyme essential oils have been shown to increase the rate of inactivation of* L. monocytogenes* and* E. coli* O157:H7 in Feta cheese [127], the cheeses being accepted by taste panellists.

## 7. Concluding Remarks and Future Trends

**Figure 1:**
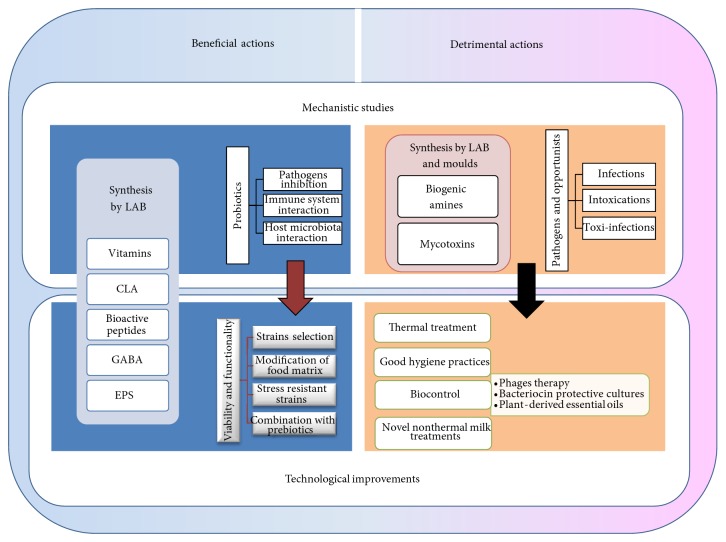
Overview of the main scientific and technological areas of interest relating microorganisms present in fermented dairy products and human health. LAB: lactic acid bacteria; CLA: conjugated linoleic acid; GABA: gamma-aminobutyric acid; EPS: exopolysaccharides.

Although the manufacture of fermented dairy products by humanity began in prehistory, we continue innovating production even today.  Figure 1 presents a schematic overview of the main areas of scientific and technological interest in relation with microorganisms present in fermented dairy products and human health.

The extraordinary recent development of next generation sequencing (NGS), functional genomics (with their related dynamic techniques such as metabolomics, proteomics, and transcriptomics), and systems biology will facilitate in the coming years a better understanding of microbial population dynamics occurring in fermented dairy products, as well as a more accurate prediction of the biochemical processes occurring in fermented milk products as depending on the microbiota which is present. Cell biology techniques are necessary tools for deciphering the interaction mechanisms between pathogens and probiotics with the host, with respect to their detrimental or beneficial action. In the case of probiotics, this knowledge will help in the selection of the best strains targeting specific human populations with defined needs. While mechanistic research advances, it is necessary to continue and improve surveillance programs of diseases caused by fermented dairy products; vigilance must remain in maintaining the hygienic conditions of dairy processing. Finally, research in new technologies providing safe alternatives to milk thermal processes, such as pasteurisation, may allow the development of safer products with organoleptic properties more to the liking of some consumers. In spite of the scientific advances, our knowledge on the effects of fermented dairy products and the accompanying microorganisms on human health remains incompletely understood.

## Figures and Tables

**Table 1:** Some examples of outbreaks caused by fermented dairy products and the pathogen involved.

Pathogen	Fermented dairy products	Outbreak details	Reference
*Brucella *	Pecorino cheese	7 cases. Made from raw milk and insufficiently aged.	[105]
*Clostridium botulinum *	Yoghurt	27 cases, 1 death. Insufficient processing of hazelnut conserve used as a flavour.	[106]
*Listeria monocytogenes *	Hard cheese	12 cases, 4 deaths. Postmanufacture contamination.	[107]
*Salmonella *	Hard cheese	Estimated 3000 cases. Cheese made from raw milk.	[108]
*Staphylococcus aureus *	Sheep milk cheese	25–27 cases. Raw milk used in production.	[109]
STECO157:H7	Gouda cheese	41 cases. Raw milk used to make cheese and numerous production/handling problems including insufficient ageing.	[110]
	Yoghurt	16 cases, 13 hospital admissions, 5 haemolytic uraemic syndrome. Possible improperly cleaned pump.	[111]

There are numerous other reports in the literature but many of them do not provide details on the dairy product involved. The table above includes data only for dairy products made with a starter culture. A more comprehensive list of outbreaks involving any kind of cheese is given elsewhere [112].

**Table 2:** Beneficial and detrimental microbial compounds that can be released in fermented dairy products during fermentation and the main producer microorganisms.

Compounds	Main producer microorganisms in dairy products	Reference
Beneficial		[11, 12, 15, 77]
Conjugated linoleic acid (CLA)	BAL (*Lactobacillus, Lactococcus, *and *Bifidobacterium*)	
Microbial exopolysaccharides (EPS)	BAL (*Lactobacillus, Lactococcus, Pediococcus*, *Streptococcus thermophilus,* and *Bifidobacterium*)	
Oligosaccharides	BAL (*Bifidobacterium and Lactobacillus*) and *Kluyveromyces lactis *	
Vitamins (B_12_, biotin, and folic acid)	BAL (*Lb. plantarum, Bifidobacterium, S. thermophilus, Lb. delbrueckii, and Propionibacterium*)	
Gamma-aminobutyric acid (GABA)	BAL (*Lactococcus, Enterococcus, Lactobacillus, Pediococcus, Streptococcus*, and* Leuconostoc*)	
Bioactive peptides:		
Immune modulatory	*Lactobacillus* GG	
Antihypertensive	*Lactobacillus *GG*, Lb. helveticus, and S. thermophilus, *	
Antimicrobial	*Lb. helveticus and Lb. acidophilus *	
Antioxidative	*Bifidobacterium longum and Lb. delbrueckii *	
Bacteriocins	BAL (*Lactococcus, Enterococcus, Lactobacillus, Pediococcus, Streptococcus, Bifidobacterium*, and* Leuconostoc*)	[101]

Detrimental		
Mycotoxins:		[28]
Aflatoxins, ochratoxin, and patulin	*Aspergillus* and *Penicillium *	
Trichothecenes, fumonisins, and zearalenone	*Fusarium *	
Biogenic Amines:		[31, 38]
Tyramine	BAL (*Enterococcus, Lb. curvatus, and Lb. brevis) *	
Putrescine	BAL (*Enterococcus, Lb. curvatus, Lb. brevis*, and* La. lactis*) and Enterobacteriaceae	
Cadaverine	Enterobacteriaceae	
Histamine	BAL (*Lb. buchner and, S. thermophilus*)	

**Table 3:** Summary of the possible mechanisms by which probiotics exert healthy effects.

Mechanisms	
(1) By inhibiting the adhesion of pathogens to the epithelium in a nonspecific manner or by competing for specific receptors and nutrients	
(2) By producing antimicrobial agents against pathogens	
(3) By inducing mucin production in the epithelial cells	
(4) By strengthening the mucosal barrier through the regeneration of epithelial cells and reduction of permeability	
(5) By modulating the immune system through the antigen-presenting cells	
(6) By inducing cytokine production from the epithelial and immune cells, resulting in enhanced cell-mediated immune responses and the activation of cytotoxic T cells, phagocytic cells, and NK cells	
(7) By increasing the proliferation of B cells through the induction of cytokines, which travel to secondary lymphatic organs in mucosa-associated lymphoid tissue and differentiate into immunoglobulin-producing plasma cells that may return to gut-associated lymphoid tissue by inducing the production of specific antibodies such as secretory IgA	

## References

[1] Quigley L., O'Sullivan O., Beresford T. P., Ross R. P., Fitzgerald G. F., Cotter P. D. (2011). Molecular approaches to analysing the microbial composition of raw milk and raw milk cheese. *International Journal of Food Microbiology*.

[2] Henri-Dubernet S., Desmasures N., Guéguen M. (2008). Diversity and dynamics of lactobacilli populations during ripening of RDO Camembert cheese. *Canadian Journal of Microbiology*.

[3] Smit G., Smit B. A., Engels W. J. M. (2005). Flavour formation by lactic acid bacteria and biochemical flavour profiling of cheese products. *FEMS Microbiology Reviews*.

[4] Facklam R. (2002). What happened to the streptococci: overview of taxonomic and nomenclature changes. *Clinical Microbiology Reviews*.

[5] Giraffa G. (2002). Enterococci from foods. *FEMS Microbiology Reviews*.

[6] Cardamone L., Quiberoni A., Mercanti D. J., Fornasari M. E., Reinheimer J. A., Guglielmotti D. M. (2011). Adventitious dairy Leuconostoc strains with interesting technological and biological properties useful for adjunct starters. *Dairy Science & Technology*.

[7] Cremonesi P., Vanoni L., Silvetti T., Morandi S., Brasca M. (2012). Identification of *Clostridium beijerinckii, Cl. butyricum, Cl. sporogenes, Cl. tyrobutyricum* isolated from silage, raw milk and hard cheese by a multiplex PCR assay. *Journal of Dairy Research*.

[8] Delbès-Paus C., Pochet S., Helinck S. (2012). Impact of Gram-negative bacteria in interaction with a complex microbial consortium on biogenic amine content and sensory characteristics of an uncooked pressed cheese. *Food Microbiology*.

[9] Delavenne E., Mounier J., Asmani K., Jany J.-L., Barbier G., Le Blay G. (2011). Fungal diversity in cow, goat and ewe milk. *International Journal of Food Microbiology*.

[10] Lavoie K., Touchette M., St-Gelais D., Labrie S. (2012). Characterization of the fungal microflora in raw milk and specialty cheeses of the province of Quebec. *Dairy Science and Technology*.

[11] Beermann C., Hartung J. (2013). Physiological properties of milk ingredients released by fermentation. *Food & Function*.

[12] Hugenholtz J., Hunik J., Santos H., Smid E. (2002). Nutraceutical production by propionibacteria. *Lait*.

[13] Lin M. Y., Young C. M. (2000). Folate levels in cultures of lactic acid bacteria. *International Dairy Journal*.

[14] Abd El-Salam M. H., El-Shafei K., Sharaf O. M., Effat B. A., Asem F. M., El-Aasar M. (2010). Screening of some potentially probiotic lactic acid bacteria for their ability to synthesis conjugated linoleic acid. *International Journal of Dairy Technology*.

[15] Hennessy A. A., Barrett E., Paul Ross R., Fitzgerald G. F., Devery R., Stanton C. (2012). The production of conjugated *α*-linolenic, *γ*-linolenic and stearidonic acids by strains of bifidobacteria and propionibacteria. *Lipids*.

[16] Penedo L. A., Nunes J. C., Gama M. A. S., Leite P. E. C., Quirico-Santos T. F., Torres A. G. (2013). Intake of butter naturally enriched with cis9,trans11 conjugated linoleic acid reduces systemic inflammatory mediators in healthy young adults. *Journal of Nutritional Biochemistry*.

[17] Chinnadurai K., Kanwal H. K., Tyagi A. K., Stanton C., Ross P. (2013). High conjugated linoleic acid enriched ghee (clarified butter) increases the antioxidant and antiatherogenic potency in female Wistar rats. *Lipids in Health and Disease*.

[18] Parvez S., Malik K. A., Ah Kang S., Kim H.-Y. (2006). Probiotics and their fermented food products are beneficial for health. *Journal of Applied Microbiology*.

[19] Hernández-Ledesma B., Del Mar Contreras M., Recio I. (2011). Antihypertensive peptides: production, bioavailability and incorporation into foods. *Advances in Colloid and Interface Science*.

[20] Moslehishad M., Ehsani M. R., Salami M. (2013). The comparative assessment of ACE-inhibitory and antioxidant activities of peptide fractions obtained from fermented camel and bovine milk by Lactobacillus rhamnosus PTCC 1637. *International Dairy Journal*.

[21] Ong L., Henriksson A., Shah N. P. (2007). Angiotensin converting enzyme-inhibitory activity in Cheddar cheeses made with the addition of probiotic *Lactobacillus casei* sp. *Lait*.

[22] Inoue K., Shirai T., Ochiai H. (2003). Blood-pressure-lowering effect of a novel fermented milk containing *γ*-aminobutyric acid (GABA) in mild hypertensives. *European Journal of Clinical Nutrition*.

[23] Padilla B., Ruiz-Matute A. I., Belloch C., Cardelle-Cobas A., Corzo N., Manzanares P. (2012). Evaluation of oligosaccharide synthesis from lactose and lactulose using *β*-galactosidases from kluyveromyces isolated from artisanal cheeses. *Journal of Agricultural and Food Chemistry*.

[24] Schwab C., Lee V., Sørensen K. I., Gänzle M. G. (2011). Production of galactooligosaccharides and heterooligosaccharides with disrupted cell extracts and whole cells of lactic acid bacteria and bifidobacteria. *International Dairy Journal*.

[25] Hidalgo-Cantabrana C., López P., Gueimonde M. (2012). Immune modulation capability of exopolysaccharides synthesised by lactic acid bacteria and bifidobacteria. *Probiotics and Antimicrobial Proteins*.

[26] Salazar N., Gueimonde M., Hernández-Barranco A. M., Ruas-Madiedo P., de Los Reyes-Gavilán C. G. (2008). Exopolysaccharides produced by intestinal Bifidobacterium strains act as fermentable substrates for human intestinal bacteria. *Applied and Environmental Microbiology*.

[27] Bernardo D., Sánchez B., Al-Hassi H. O. (2012). Microbiota/host crosstalk biomarkers: regulatory response of human intestinal dendritic cells exposed to *Lactobacillus* extracellular encrypted peptide. *PLoS ONE*.

[28] Marasas W. F. O., Gelderblom W. C. A., Vismer H. F., Leslie J. F., Bandyopadhayay R., Visconti A. (2008). Mycotoxins: a gobal problem. *Mycotoxins*.

[29] Keller N. P., Turner G., Bennett J. W. (2005). Fungal secondary metabolism—from biochemistry to genomics. *Nature Reviews Microbiology*.

[30] Prandini A., Tansini G., Sigolo S., Filippi L., Laporta M., Piva G. (2009). On the occurrence of aflatoxin M1 in milk and dairy products. *Food and Chemical Toxicology*.

[31] Linares D. M., Martĺn M. C., Ladero V., Alvarez M. A., Fernández M. (2011). Biogenic amines in dairy products. *Critical Reviews in Food Science and Nutrition*.

[32] Ten Brink B., Damink C., Joosten H. M. L. J., Huis in 't Veld J. H. J. (1990). Occurrence and formation of biologically active amines in foods. *International Journal of Food Microbiology*.

[33] Pircher A., Bauer F., Paulsen P. (2007). Formation of cadaverine, histamine, putrescine and tyramine by bacteria isolated from meat, fermented sausages and cheeses. *European Food Research and Technology*.

[34] Marino M., Maifreni M., Moret S., Rondinini G. (2000). The capacity of *Enterobacteriaceae* species to produce biogenic amines in cheese. *Letters in Applied Microbiology*.

[35] Fernández M., Linares D. M., Alvarez M. A. (2004). Sequencing of the tyrosine decarboxylase cluster of *Lactococcus lactis* IPLA 655 and the development of a PCR method for detecting tyrosine decarboxylating lactic bacteria. *Journal of Food Protection*.

[36] Cruz Martín M., Fernández M., Linares D. M., Alvarez M. A. (2005). Sequencing, characterization and transcriptional analysis of the histidine decarboxylase operon of *Lactobacillus buchneri*. *Microbiology*.

[37] Bonetta S., Carraro E., Coïsson J. D., Travaglia F., Arlorio M. (2008). Detection of biogenic amine producer bacteria in a typical Italian goat cheese. *Journal of Food Protection*.

[38] Calles-Enríquez M., Ladero V., Fernández M., Martín M. C., Alvarez M. A. (2010). Extraction of RNA from fermented milk products for *in situ* gene expression analysis. *Analytical Biochemistry*.

[39] Ladero V., Rattray F. P., Mayo B., Martín M. C., Fernández M., Alvarez M. A. (2011). Sequencing and transcriptional analysis of the biosynthesis gene *cluster* of putrescine-producing *Lactococcus lactis*. *Applied and Environmental Microbiology*.

[40] Linares D. M., del Río B., Ladero V. (2012). Factors influencing biogenic amines accumulation in dairy products. *Frontiers in Microbiology*.

[41] Fernández M., Del Río B., Linares D. M., Martín M. C., Alvarez M. A. (2006). Real-time polymerase chain reaction for quantitative detection of histamine-producing bacteria: use in cheese production. *Journal of Dairy Science*.

[42] Roig-Sagués A. X., Molina A. P., Hernández-Herrero M. M. (2002). Histamine and tyramine-forming microorganisms in Spanish traditional cheeses. *European Food Research and Technology*.

[43] Martínez N., Martín M. C., Herrero A., Fernández M., Alvarez M. A., Ladero V. (2011). QPCR as a powerful tool for microbial food spoilage quantification: significance for food quality. *Trends in Food Science and Technology*.

[44] Redruello B., Ladero V., Cuesta I. (2013). A fast, reliable, ultra high performance liquid chromatography method for the simultaneous determination of amino acids, biogenic amines and ammonium ions in cheese, using diethyl ethoxymethylenemalonate as a derivatising agent. *Food Chemistry*.

[45] FAO-WHO (2006). Probiotics in food. Health and nutritional properties and guidelines for 339 evaluation. *FAO Food and Nutritional Paper*.

[46] Gueimonde M., de los Reyes-Gavilán C. G., Sánchez B., Salminen S., von Wright A., Ouwehand A., Lahtinen S. (2011). Stability of lactic acid bacteria in foods and supplements. *Lactic Acid Bacteria, Microbiological and Functional Aspects*.

[47] Lehtoranta L. (2012). *Probiotics and virus infections: The effects of Lactobacillus rhamnosus GG on respiratory and gastrointestinal virus infections [Ph.D. thesis]*.

[48] Power S. E., O'Toole P. W., Stanton C., Ross R. P., Fitzgerald G. F. (2014). Intestinal microbiota, diet and health. *British Journal of Nutrition*.

[49] Salminen S., Nybom S., Meriluoto J., Collado M. C., Vesterlund S., El-Nezami H. (2010). Interaction of probiotics and pathogens-benefits to human health?. *Current Opinion in Biotechnology*.

[50] Botić T., Klingberg T. D., Weingartl H., Cencič A. (2007). A novel eukaryotic cell culture model to study antiviral activity of potential probiotic bacteria. *International Journal of Food Microbiology*.

[51] Juntunen M., Kirjavainen P. V., Ouwehand A. C., Salminen S. J., Isolauri E. (2001). Adherence of probiotic bacteria to human intestinal mucus in healthy infants and during rotavirus infection. *Clinical and Diagnostic Laboratory Immunology*.

[52] Ouwehand A. C., Tuomola E. M., Tölkkö S., Salminen S. (2001). Assessment of adhesion properties of novel probiotic strains to human intestinal mucus. *International Journal of Food Microbiology*.

[53] Ouwehand A. C., Suomalainen T., Tölkkö S., Salminen S. (2002). In vitro adhesion of propionic acid bacteria to human intestinal mucus. *Lait*.

[54] Deplancke B., Gaskins H. R. (2001). Microbial modulation of innate defense: goblet cells and the intestinal mucus layer. *The American Journal of Clinical Nutrition*.

[55] Banasaz M., Norin E., Holma R., Midtvedt T. (2002). Increased enterocyte production in gnotobiotic rats mono-associated with *Lactobacillus rhamnosus* GG. *Applied and Environmental Microbiology*.

[56] Pipenbaher N., Moeller P. L., Dolinšek J., Jakobsen M., Weingartl H., Cencič A. (2009). Nitric oxide (NO) production in mammalian non-tumorigenic epithelial cells of the small intestine and macrophages induced by individual strains of lactobacilli and bifidobacteria. *International Dairy Journal*.

[57] Yan F., Cao H., Cover T. L., Whitehead R., Washington M. K., Polk D. B. (2007). Soluble proteins produced by probiotic bacteria regulate intestinal epithelial cell survival and growth. *Gastroenterology*.

[58] Isolauri E., Majamaa H., Arvola T., Rantala I., Virtanen E., Arvilommi H. (1993). *Lactobacillus casei* strain GG reverses increased intestinal permeability induced by cow milk in suckling rats. *Gastroenterology*.

[59] Otte J.-M., Podolsky D. K. (2004). Functional modulation of enterocytes by gram-positive and gram-negative microorganisms. *The American Journal of Physiology—Gastrointestinal and Liver Physiology*.

[60] Miettinen M., Matikainen S., Vuopio-Varkila J. (1998). Lactobacilli and streptococci induce interleukin-12 (IL-12), IL-18, and gamma interferon production in human peripheral blood mononuclear cells. *Infection and Immunity*.

[61] Vinderola G., Matar C., Perdigon G. (2005). Role of intestinal epithelial cells in immune effects mediated by gram-positive probiotic bacteria: involvement of toll-like receptors. *Clinical and Diagnostic Laboratory Immunology*.

[62] Foligne B., Nutten S., Grangette C. (2007). Correlation between *in vitro* and *in vivo* immunomodulatory properties of lactic acid bacteria. *World Journal of Gastroenterology*.

[63] Miettinen M., Veckman V., Latvala S., Sareneva T., Matikainen S., Julkunen I. (2008). Live *Lactobacillus* rhamnosus and *Streptococcus pyogenes* differentially regulate Toll-like receptor (TLR) gene expression in human primary macrophages. *Journal of Leukocyte Biology*.

[64] O'Hara A. M., O'Regan P., Fanning Á. (2006). Functional modulation of human intestinal epithelial cell responses by *Bifidobacterium infantis* and *Lactobacillus salivarius*. *Immunology*.

[65] Miettinen M., Lehtonen A., Julkunen I., Matikainen S. (2000). *Lactobacilli* and *Streptococci* activate NF-*κ*B and STAT signaling pathways in human macrophages. *The Journal of Immunology*.

[66] Veckman V., Miettinen M., Matikainen S. (2003). Lactobacilli and streptococci induce inflammatory chemokine production in human macrophages that stimulates Th1 cell chemotaxis. *Journal of Leukocyte Biology*.

[67] Veckman V., Miettinen M., Pirhonen J., Sirén J., Matikainen S., Julkunen I. (2004). *Streptococcus pyogenes* and *Lactobacillus rhamnosus* differentially induce maturation and production of Th1-type cytokines and chemokines in human monocyte-derived dendritic cells. *Journal of Leukocyte Biology*.

[68] Latvala S., Miettinen M., Kekkonen R. (2009). Potentially probiotic bacteria induce cytokine production and suppressor of cytokine signaling 3 gene expression in human monocyte-derived macrophages. *Cytokine*.

[69] Latvala S., Miettinen M., Kekkonen R. A., Korpela R., Julkunen I. (2011). *Lactobacillus rhamnosus* GG and *Streptococcus thermophilus* induce suppressor of cytokine signalling 3 (SOCS3) gene expression directly and indirectly via interleukin-10 in human primary macrophages. *Clinical and Experimental Immunology*.

[70] Weiss G., Christensen H. R., Zeuthen L. H., Vogensen F. K., Jakobsen M., Frøkiær H. (2011). Lactobacilli and bifidobacteria induce differential interferon-*β* profiles in dendritic cells. *Cytokine*.

[71] Ventola H., Lehtoranta L., Madetoja M. (2012). Effects of the viability of *Lactobacillus rhamnosus* GG on rotavirus infection in neonatal rats. *World Journal of Gastroenterology*.

[72] Vinderola C. G., Gueimonde M., Delgado T., Reinheimer J. A., Reyes-Gavilán C. G. D. L. (2000). Characteristics of carbonated fermented milk and survival of probiotic bacteria. *International Dairy Journal*.

[73] Kneifel W., Jaros D., Erhard F. (1993). Microflora and acidification properties of yogurt and yogurt-related products fermented with commercially available starter cultures. *International Journal of Food Microbiology*.

[74] Antunes A. E. C., Cazetto T. F., Abolini H. M. (2005). Viability of probiotic micro-organisms during storage, postacidification and sensory analysis of fat-free yogurts with added whey protein concentrate. *International Journal of Dairy Technology*.

[75] Gueimonde M., de Los Reyes-Gavilán C. G. (2004). Reduction of incubation time in carbonated *Streptococcus thermophilus*/*Lactobacillus acidophilus* fermented milks as affected by the growth and acidification capacity of the starter strains. *Milchwissenschaft*.

[76] Gaudreau H., Champagne C. P., Remondetto G. E., Bazinet L., Subirade M. (2013). Effect of catechins on the growth of oxygen-sensitive probiotic bacteria. *Food Research International*.

[77] Mozzi F., Gerbino E., Font De Valdez G., Torino M. I. (2009). Functionality of exopolysaccharides produced by lactic acid bacteria in an *in vitro* gastric system. *Journal of Applied Microbiology*.

[78] Ramchandran L., Shah N. P. (2010). Characterization of functional, biochemical and textural properties of synbiotic low-fat yogurts during refrigerated storage. *LWT: Food Science and Technology*.

[79] Ruiz L., Ruas-Madiedo P., Gueimonde M., De Los Reyes-Gavilán C. G., Margolles A., Sánchez B. (2011). How do bifidobacteria counteract environmental challenges? Mechanisms involved and physiological consequences. *Genes and Nutrition*.

[80] Gueimonde M., Noriega L., Margolles A., De Los Reyes-Gavilan C. G., Salminen S. (2005). Ability of *Bifidobacterium* strains with acquired resistance to bile to adhere to human intestinal mucus. *International Journal of Food Microbiology*.

[81] de los Reyes-Gavilán C. G., Suárez A., Fernández-García M., Margolles A., Gueimonde M., Ruas-Madiedo P. (2011). Adhesion of bile-adapted *Bifidobacterium* strains to the HT29-MTX cell line is modified after sequential gastrointestinal challenge simulated in vitro using human gastric and duodenal juices. *Research in Microbiology*.

[82] Sánchez B., Fernández-García M., Margolles A., de los Reyes-Gavilán C. G., Ruas-Madiedo P. (2010). Technological and probiotic selection criteria of a bile-adapted *Bifidobacterium animalis* subsp. *lactis* strain. *International Dairy Journal*.

[83] Sheehan V. M., Sleator R. D., Fitzgerald G. F., Hill C. (2006). Heterologous expression of BetL, a betaine uptake system, enhances the stress tolerance of *Lactobacillus salivarius* UCC118. *Applied and Environmental Microbiology*.

[84] Sheehan V. M., Sleator R. D., Hill C., Fitzgerald G. F. (2007). Improving gastric transit, gastrointestinal persistence and therapeutic efficacy of the probiotic strain *Bifidobacterium breve* UCC2003. *Microbiology*.

[85] ISAPP 6th Meeting of the International Scientific Association of Probiotics and Prebiotics, London, Ontario, Canada. http://www.isapp.net/Portals/0/docs/Annual%20Meetings/2008/2008_ISAPP_Meeting_Report.pdf.

[86] Koh J. H., Choi S. H., Park S. W., Choi N.-J., Kim Y., Kim S. H. (2013). Synbiotic impact of tagatose on viability of *Lactobacillus rhamnosus* strain GG mediated by the phosphotransferase system (PTS). *Food Microbiology*.

[87] Riaz Q. U. A., Masud T. (2013). Recent trends and applications of encapsulating materials for probiotic stability. *Critical Reviews in Food Science and Nutrition*.

[88] de Santis E. P. L., Foddai A., Virdis S., Marongiu P., Pilo A. L., Scarano C. (2008). Toxin gene pattern in *Bacillus cereus* group strains isolated from sheep ricotta cheese. *Veterinary Research Communications*.

[89] Coton M., Delbés-Paus C., Irlinger F. (2012). Diversity and assessment of potential risk factors of Gram-negative isolates associated with French cheeses. *Food Microbiology*.

[90] Gould L. H., Walsh K. A., Vieira A. R. (2013). Surveillance for foodborne disease outbreaks—united States, 1998–2008. *Morbidity and Mortality Weekly Report*.

[91] Centers for Disease Control and Prevention (2013). Surveillance for foodborne disease outbreaks-United States, 2009-2010. *Morbidity and Mortality Weekly Reports*.

[92] European Food Safety Authority (2012). The European Union summary report on trends and sources of zoonoses, zoonotic agents and food-borne outbreaks in 2012. *EFSA Journal*.

[93] Naldini M. C. M., Viotto W. H., Kuaye A. Y. (2009). Behaviour of *Listeria monocytogenes* inoculated into Minas Frescal cheese made by direct acidification or lactic culture during refrigerated storage. *International Journal of Dairy Technology*.

[94] Cartwright E. J., Jackson K. A., Johnson S. D., Graves L. M., Silk B. J., Mahon B. E. (2013). Listeriosis outbreaks and associated food vehicles, United States, 1998–2008. *Emerging Infectious Diseases*.

[95] LeJeune J. T., Rajala-Schultz P. J. (2009). Unpasteurized milk: a continued public health threat. *Clinical Infectious Diseases*.

[96] Allerberger F., Wagner M. (2010). Listeriosis: a resurgent foodborne infection. *Clinical Microbiology and Infection*.

[97] Farrokh C., Jordan K., Auvray F. (2013). Review of Shiga-toxin-producing *Escherichia coli* (STEC) and their significance in dairy production. *International Journal of Food Microbiology*.

[98] Hennekinne J.-A., De Buyser M.-L., Dragacci S. (2012). *Staphylococcus aureus* and its food poisoning toxins: characterization and outbreak investigation. *FEMS Microbiology Reviews*.

[99] Peck M. W. (2006). *Clostridium botulinum* and the safety of minimally heated, chilled foods: an emerging issue?. *Journal of Applied Microbiology*.

[100] Scallan E., Hoekstra R. M., Angulo F. J. (2011). Foodborne illness acquired in the United States—major pathogens. *Emerging Infectious Diseases*.

[101] Molloy E. M., Hill C., Cotter P. D., Fuquay J., Fox P., McSweeney P. (2011). Bacteriocins. *Encyclopedia of Dairy Sciences*.

[102] Donnelly C. W., Fox P. F., McSweeney P. L. H., Cogan T. M., Guinee T. P. (2004). Growth and survival of microbial pathogens in cheese. *Cheese: Chemistry, Physics and Microbiology*.

[103] de Buyser M.-L., Dufour B., Maire M., Lafarge V. (2001). Implication of milk and milk products in food-borne diseases in France and in different industrialised countries. *International Journal of Food Microbiology*.

[104] Langer A. J., Ayers T., Grass J., Lynch M., Angulo F. J., Mahon B. E. (2012). Nonpasteurized dairy products, disease outbreaks, and State Laws-United States, 1993–2006. *Emerging Infectious Diseases*.

[105] Galbraith N. S., Ross M. S., de Mowbray R. R., Payne D. J. (1969). Outbreak of *Brucella melitensis* type 2 infection in London. *British Medical Journal*.

[106] O'Mahony M., Mitchell E., Gilbert R. J. (1990). An outbreak of foodborne botulism associated with contaminated hazelnut yoghurt. *Epidemiology and Infection*.

[107] Yde M., Naranjo M., Mattheus W. (2012). Usefulness of the European epidemic intelligence information system in the management of an outbreak of listeriosis, Belgium, 2011. *Eurosurveillance*.

[108] van Duynhoven Y. T. H. P., Isken L. D., Borgen K. (2009). A prolonged outbreak of *Salmonella* Typhimurium infection related to an uncommon vehicle: hard cheese made from raw milk. *Epidemiology & Infection*.

[109] Bone F. J., Bogie D., Morgan-Jones S. C. (1989). Staphylococcal food poisoning from sheep milk cheese. *Epidemiology and Infection*.

[110] McCollum J. T., Williams N. J., Beam S. W. (2012). Multistate outbreak of *Escherichia coli* O157:H7 infections associated with in-store sampling of an aged raw-milk Gouda cheese, 2010. *Journal of Food Protection*.

[111] Morgan D., Newman C. P., Hutchinson D. N., Walker A. M., Rowe B., Majid F. (1993). Verotoxin producing *Escherichia coli* O 157 infections associated with the consumption of yoghurt. *Epidemiology and Infection*.

[112] Kousta M., Mataragas M., Skandamis P., Drosinos E. H. (2010). Prevalence and sources of cheese contamination with pathogens at farm and processing levels. *Food Control*.

[113] D'Amico D. J., Druart M. J., Donnelly C. W. (2008). 60-day aging requirement does not ensure safety of surface-mold-ripened soft cheeses manufactured from raw or pasteurized milk when Listeria monocytogenes is introduced as a postprocessing contaminant. *Journal of Food Protection*.

[114] Schlesser J. E., Gerdes R., Ravishankar S., Madsen K., Mowbray J., Teo A. Y.-L. (2006). Survival of a five-strain cocktail of *Escherichia coli* O157:H7 during the 60-day aging period of cheddar cheese made from unpasteurized milk. *Journal of Food Protection*.

[115] Sango D. M., Abela D., Mcelhatton A., Valdramidis V. P. (2014). Assisted ultrasound applications for the production of safe foods. *Journal of Applied Microbiology*.

[116] Deeth H. C., Datta N., Fuquay J. W., Fox P., McSweeney P. (2011). Non-thermal technologies: pulsed electic field technology and ultrasonication. *Encyclopedia of Dairy Sciences*.

[117] Chawla R., Patil G. R., Singh A. K. (2011). High hydrostatic pressure technology in dairy processing: a review. *Journal of Food Science and Technology*.

[118] López-Pedemonte T., Roig-Sagués A. X., Lamo S. D., Gervilla R., Guamis B. (2007). High hydrostatic pressure treatment applied to model cheeses made from cow's milk inoculated with Staphylococcus aureus. *Food Control*.

[119] Modi R., Hirvi Y., Hill A., Griffiths M. W. (2001). Effect of phage on survival of *Salmonella* Enteritidis during manufacture and storage of Cheddar cheese made from raw and pasteurized milk. *Journal of Food Protection*.

[120] Bueno E., García P., Martínez B., Rodríguez A. (2012). Phage inactivation of *Staphylococcus aureus* in fresh and hard-type cheeses. *International Journal of Food Microbiology*.

[121] Tomat D., Mercanti D., Balagué C., Quiberoni A. (2013). Phage biocontrol of enteropathogenic and shiga toxin-producing *Escherichia coli* during milk fermentation. *Letters in Applied Microbiology*.

[122] Carlton R. M., Noordman W. H., Biswas B., De Meester E. D., Loessner M. J. (2005). Bacteriophage P100 for control of *Listeria monocytogenes* in foods: genome sequence, bioinformatic analyses, oral toxicity study, and application. *Regulatory Toxicology and Pharmacology*.

[123] Schellekens M. M., Woutersi J., Hagens S., Hugenholtz J. (2007). Bacteriophage P100 application to control *Listeria monocytogenes* on smeared cheese. *Milchwissenschaft*.

[124] Varalakshmi S., Balasubramanyam B. V., Surendranath B., Bagath M., Rajendran D. (2014). Use of novel lactic acid bacterial strains with antagonistic activity for the preparation of safe indigenous fermented dairy foods (*Dahi* and *Raita*). *Journal of Food Safety*.

[125] García P., Rodríguez L., Rodríguez A., Martínez B. (2010). Food biopreservation: promising strategies using bacteriocins, bacteriophages and endolysins. *Trends in Food Science and Technology*.

[126] Rodriguez E., Arques J. L., Nuñez M., Gaya P., Medina M. (2005). Combined effect of high-pressure treatments and bacteriocin-producing lactic acid bacteria on inactivation of *Escherichia coli* O157:H7 in raw-milk cheese. *Applied and Environmental Microbiology*.

[127] Govaris A., Botsoglou E., Sergelidis D., Chatzopoulou P. S. (2011). Antibacterial activity of oregano and thyme essential oils against *Listeria monocytogenes* and *Escherichia coli* O157:H7 in feta cheese packaged under modified atmosphere. *Food Science and Technology*.

